# Redox Signaling via Oxidative Inactivation of PTEN Modulates Pressure-Dependent Myogenic Tone in Rat Middle Cerebral Arteries

**DOI:** 10.1371/journal.pone.0068498

**Published:** 2013-07-05

**Authors:** Debebe Gebremedhin, Maia Terashvili, Nadi Wickramasekera, David X. Zhang, Nicole Rau, Hiroto Miura, David R. Harder

**Affiliations:** 1 Department of Physiology, Medical College of Wisconsin, Milwaukee, Wisconsin, United States of America; 2 Department of Medicine, Medical College of Wisconsin, Milwaukee, Wisconsin, United States of America; 3 Cardiovascular Research Center, Medical College of Wisconsin, Milwaukee, Wisconsin, United States of America; 4 Clement Zablocki VA Medical Center, Milwaukee, Wisconsin, United States of America; Massachusetts General Hospital/Harvard Medical School, United States of America

## Abstract

The present study examined the level of generation of reactive oxygen species (ROS) and roles of inactivation of the phosphatase PTEN and the PI3K/Akt signaling pathway in response to an increase in intramural pressure-induced myogenic cerebral arterial constriction. Step increases in intraluminal pressure of cannulated cerebral arteries induced myogenic constriction and concomitant formation of superoxide (O_2_^.−^) and its dismutation product hydrogen peroxide (H_2_O_2_) as determined by fluorescent HPLC analysis, microscopic analysis of intensity of dihydroethidium fluorescence and attenuation of pressure-induced myogenic constriction by pretreatment with the ROS scavenger 4,hydroxyl-2,2,6,6-tetramethylpiperidine1-oxyl (tempol) or Mito-tempol or MitoQ in the presence or absence of PEG-catalase. An increase in intraluminal pressure induced oxidation of PTEN and activation of Akt. Pharmacological inhibition of endogenous PTEN activity potentiated pressure-dependent myogenic constriction and caused a reduction in NPo of a 238 pS arterial K_Ca_ channel current and an increase in [Ca^2+^]_i_ level in freshly isolated cerebral arterial muscle cells (CAMCs), responses that were attenuated by Inhibition of the PI3K/Akt pathway. These findings demonstrate an increase in intraluminal pressure induced increase in ROS production triggered redox-sensitive signaling mechanism emanating from the cross-talk between oxidative inactivation of PTEN and activation of the PI3K/Akt signaling pathway that involves in the regulation of pressure-dependent myogenic cerebral arterial constriction.

## Introduction

The pressure-dependent myogenic constriction of arteries that occurs independent of neuronal activity has long been considered as an intrinsic functional behavior of the arterial muscle [1], [2]. The brain is one of the organs relying upon intrinsic or myogenic mechanisms developing within blood vessels in response to stretch or transmural pressure and is critical for maintenance of steady state cerebral blood flow (CBF) [2]. Reactive oxygen species (ROS) could be generated under physiological conditions and serve as molecules signaling normal tissue functions, whereas their rate of production is altered or increased under oxidative stress and in a variety of pathological conditions contributing to altered organ function and tissue damage. Despite existing evidence for stretch- or an increase in intraluminal pressure-induced increased ROS generation [3], [4], [5] very little is known about its impact on signaling mechanisms modulating the development and maintenance of pressure-induced cerebral arterial myogenic constriction.

The maintenance of steady state distribution of cerebral blood flow effected through myogenic tone development is critically important for neuronal cells as they do not store glucose and depend on a continuous blood supply of glucose and oxygen for normal use or in conditions of increased metabolic demand [6], [7], [8], [9], [10]. An increase in intraluminal pressure-induced depolarization of vascular smooth muscle and vasoconstriction has been previously described in the middle cerebral artery [6]. Albeit the signaling mechanisms are yet to be completely understood, this was an initial classic observation that identified an intrinsic electromechanical coupling through which cerebral blood flow could be maintained in the face of changing intravascular pressure within an enclosed space. In this study we attempt to extend this work by determining the effects of increasing intravascular pressure on ROS generation and examining role of a potential redox-sensitive signaling mechanism involving the phosphatase and tensin homolog deleted on chromosome ten (PTEN) that could be implicated in the modulation of the mechanisms crucial for the development of pressure-induced myogenic response under physiological or pathophysiological conditions.

Phosphatidylinositol 3-kinase (PI3K) is one of the endogenous signaling pathways sensitive to modulation by ROS, specifically superoxide (O_2_^–^) and hydrogen peroxide (H_2_O_2_), and is well recognized in signaling the effects of mechanical forces including pressure, shear force and stretch on the vascular muscle cell and in other cell types [11], [12]. The dual phosphatase PTEN is a tumor suppressor gene that functions as a pivotal signaling molecule regulating the phosphorylated state of a variety of molecules linked to activation of phosphoinositde-3-kiase (PI3K) and increased production of the downstream kinase Akt [13], [14].

The present studies were designed to investigate the hypothesis that an increase in intraluminal pressure induced generation of the ROS O_2_^–^ or H_2_O_2_ is associated with oxidation of the dual phosphatase PTEN that could result in reduced open state probability of a 238 pS K_Ca_ single-channel current, increased [Ca^2+^]_i_ in isolated cerebral arterial muscle cells and enhanced pressure-dependent myogenic cerebral arterial constriction that could be linked to an increased activity of the PI3K/Akt signaling pathway.

## Materials and Methods

### Drugs and Chemicals

4, hydroxyl-2,2,6,6-tetramethylpiperidine 1-oxyl (tempol) and PEG-catalase were obtained from Sigma. A mitochondrial-targeted form of tempol (Mito-tempol, MT) and MitoQ were synthesized and prepared by Dr. Joy Joseph (Free Radical Center, Medical College of Wisconsin, Milwaukee, WI). All compounds were dissolved in physiological salt solution (PSS) and kept in the dark. DHE, PTEN, Akt and phospho-Akt antibodies were from Cell Signaling Technology (Danvers, MA). Potassium *Bisperoxo*(1,10-phenanthroline) oxovanadate (bpV(phen) and potassium *Bisperoxo*(1,10-picolinato) oxovanadate (V) (bpV(pic) were obtained from EMD Millipore (Billerica, MA), ZSTK474, PI3K inhibitor was obtained from SelleckBio (Houston, TX).

### Pressure-induced Myogenic Constriction of Cerebral Arteries

The animal protocols used in this study were approved by the Medical College of Wisconsin Institutional Animal Care and Use Committee (I.D. = AUA0001008). Isolation and cannulation of rat middle cerebral arteries (referred to as cerebral arteries henceforth) for measurement of myogenic response were performed as previously described [6] Briefly, male Sprague-Dawley rats (10–12 weeks) were anesthetized with sodium pentobarbital (100 mg/kg, i.p.) and decapitated after the disappearance of corneal reflexes. Brains were quickly removed and placed on a dissecting dish containing ice-cold PSS of the following composition (mM): NaCl (141), KCl (4.7), CaCl_2_ (2.5), MgCl_2_ (0.72), KH_2_PO_4_ (1.7), NaHCO_3_ (25), glucose (11) and HEPES (5). The pH was maintained between 7.4–7.5 by bubbling with 95% O_2_ and 5% CO_2_ and checked at the beginning of each experiment using a blood gas analyzer (ABL80 FLEX, Radiometer Medical ApS, Denmark). Cerebral arterial segments were carefully dissected out of the brain under a dissecting microscope and placed in PSS in a dish. Both ends of the isolated cerebral arterial segments were cannulated on glass pipettes in a pressure myograph and tied in place with 8–0 sutures. The internal diameter of the cannulated cerebral arterial segments was measured using a Living Systems Video Dimension Analyzer, chamber and pressure servo control (Systems Instrumentation, Burlington, VT, USA). The isolated cerebral arterial segments, denuded of the endothelium by brief passage of bolus of air for 1–2 min, were pressurized at 40 mm Hg and equilibrated for 30–60 min while being superfused with PSS and bubbled with a mixture of 95% O_2_ and 5% CO_2_ until the arterial segments constricted to 40–50% of their original diameter. All vessels not gaining spontaneous tone were excluded from the study. The intraluminal pressure was then increased in steps of 20 mm Hg between 20 mm Hg and 120 mm Hg and inner diameter was measured every 1 and 5 minutes at each pressure level. All experimental drugs were applied to the bath at the specified concentrations and time of incubation prior to repeat of measurement of pressure-induced changes in inner vessel diameter.

### Fluorescent Detection of Pressure-induced Superoxide Production

To determine the intraluminal pressure-dependent increase in O_2_^–^ production, isolated and cannulated cerebral arterial segments of the rat were treated with the superoxide detecting fluorescent probe dihydroethidine (DHE, 1 µM; Molecular Probes) in the absence or presence of the specific peptide inhibitor of NADPH-oxidase gp91-ds-tat (5 µM) or its control scrambled gp91 sequence (gp91-scramb-tat, 5 µM), or with the mitochondrial antioxidant MitoQ (1 µM) and allowed to equilibrate at a resting intraluminal pressure of 40 mm Hg for 20 min at 37°C in dark. The cerebral arterial segments were then pressurized from 40 mm Hg to 100 or 120 mm Hg for 60 min, and then removed from the chamber, rinsed in PSS and placed on a microscope slide in a spacer between the slide and cover slip in dark to begin taking fluorescent images of the arterial segments to determine the production of superoxide by fluorescent microscopy using a Nikon E-600 fluorescent microscope equipped with TRITC (tetramethyl rhodamine iso-thiocyanate) filters with excitation at 510 nm and emission at 595 nm [15], [16]. Fluorescence signal was quantified by measuring the mean pixel value (*F*) of a manually selected area of a frame of a vessel image using MetaMorph software (Molecular Devices). The values were exported to Microsoft Excel and the fluorescence change (*ΔF) = F–BF* was computed, where *B*F is the mean of the background fluorescence signals.

### Measurement of Pressure-induced Intracellular Superoxide Generation by HPLC

Freshly isolated and cannulated rat cerebral arterial segments maintained at 40 mm Hg were first treated in dark with the superoxide detecting fluorescent probe dihydroethidine (DHE, 1 µM), and then pressurized to 120 mm Hg for 60 min at 37°C, and then washed twice with PSS in dark. The cerebral arterial segments were then lysed in 0.25 ml lyses buffer (0.1% Triton X-100 in D-PBS, pH 7.4), and 50 µl of the cell lysate was used to determine cell protein level as described previously [15], [16] The remaining lysate solution was mixed with 0.5 ml of n-butanol and extracted in dark by vortexing for 1 min followed by centrifugation at 10,000×g for 10 min at 24°C as previously described [15], [16]. The n-butanol phase was separated and evaporated under stream of nitrogen gas. The dried samples were taken up by adding 0.1 ml of HPLC grade water. The HE, ethidium (E^+^) and the superoxide oxidation product of DHE 2-hydroxyethidium (2-OH-E^+^) were separated using an Agilent 1100 system equipped with UV-visible absorption and fluorescence detectors. To obtain UHPLC-like conditions, a fused core C18 column (Phenomenex, Kinetex C18, 100×4.6 mm, 2.6 µm) was used. Typically, a gradient elution using an aqueous mobile phase with increasing fraction of acetonitrile (from 10–20% to 100% over 5 min) in the presence of 0.1% trifluoroacetic acid (TFA) was used. The analytes were eluted using a flow rate of 1.5 ml/min. Mass spectra were obtained using a 7.0-tesla Fourier transform ion cyclotron resonance mass spectrometer, interfaced with an Agilent 1100 HPLC system as previously described [17]. The HPLC peak area for each experiment was normalized to protein concentration of the sample and compared as previously described [15], [16], [18].

### Western Blotting

Following pressurization of the cannulated cerebral arterial segments at 40 mm Hg or 120 m Hg for 60 min, the cerebral arterial segments were homogenized using a Potter-Elvehjem tissue grinder with a Teflon pestle in ice-cold lysate buffer (20 mM Tris-HCl, 150 mM NaCl, 1 mM EDTA, 1 mM EGTA, 0.01% Triton X-100, 10 mM N-methylmaleimide (NEM), protease and phosphatase inhibitors). The homogenate was centrifuged at 4°C for 15 min at 12 000×g. Supernatants were then removed and protein levels were quantitated from each sample (Protein assay from BioRad Lab., Hercules, CA). The soluble fractions were loaded at equal protein concentrations (25 µg per lane). The proteins were separated in a 10% Tris-glycine gel (BioRad Lab., Hercules, CA) and then electrophoretically transferred to PVDF membrane (Millipore Corporation, Bedford, MA) at 100 V for 1h. The blot was incubated in TBS buffer containing 0.1% Tween-20 and 10% nonfat dry milk for 1 h at room temperature to block nonspecific binding sites and then incubated overnight at 4°C with the primary antibody specific for PTEN, Phospho-PTEN, Akt and Phospho-Akt, (Cell Signaling) at 1∶1000 dilutions. Antibody against β-actin (Sigma, 1∶3000 dilutions) was used to serve as an internal control. The blot was washed with TBS containing 0.1% Tween-20 and then incubated with Goat-anti-rabbit antibody (BioRad Lab., Hercules, CA) at 1∶2000 dilution followed by additional washes before developing with ECL Plus reagent (Amersham Biosciences, UK). Blot was exposed to Hyperfilm ECL from Amersham Biosciences.

### Enzymatic Dispersion of Cerebral Arterial Segments to Single Cells

Freshly isolated rat cerebral arterial segments were placed in separate vials containing 10 ml solution of bovine serum albumin (0.5 mg/ml) in low-calcium arterial muscle cell dissociation solution composed of (in mM): 134 NaCl, 5.2 KCl, 1.2 MgSO_4_.7 H_2_O, 1.18 KH_2_PO_4_, 0.05 CaCl_2_, 24 NaHCO_3_, 11 glucose, and 10 N-2-hydroxyethylpiperazine-N′-2-ethanesulfonic acid (HEPES) (pH = 7.4), for 10 min at room temperature. These arterial segments were then transferred to another vial containing 1 ml solution of dithiothreitol (Sigma, St Louis, MO, USA) (0.5 mg/ml) and papain (Worthington, Freehold, NJ, USA) (60 Units/ml) in low-calcium dissociation solution, and placed in a water-jacketed beaker (at 37°C) and incubated for 20 min. After incubation, the supernatant layer was removed and replaced with 0.5 ml of dissociation solution containing collagenase type II (240 Units/ml; Worthington, Freehold, NJ, USA), elastase (15 Units/ml), and trypsin inhibitor (0.1 mg/ml, Sigma, St Louis, MO, USA), and incubated at 37°C (pH = 7.4). Supernatant fractions were collected at 5 min intervals and each fraction was diluted to 10 ml with fresh low-calcium dissociation solution. The procedure was repeated by incubating the remaining cerebral arterial tissue with fresh enzyme containing low-calcium dissociation solution. Complete dispersion of the cerebral arterial segments to single smooth muscle cells was typically attained within 30–40 min of incubation. The dissociated cerebral arterial smooth muscle cells were kept in a refrigerator at 4°C for a maximum of six hours and used for fluorometric intracellular calcium measurements and for patch clamp recordings of single-channel K_Ca_ currents in the present study.

### Single-channel K_Ca_ Current Recordings

Single-channel K_Ca_ currents were recorded at room temperature from cell-attached patches of dissociated smooth muscle cells of freshly isolated rat cerebral arteries using the patch clamp technique as previously described [15], [19], [20]. Briefly, recording pipettes were fabricated from borosilicate glass pulled on a 2-stage micropipette puller (PC-84) and heat polished under a microscope (Narishige MF-83 heat polisher). The recording pipettes were mounted on a three-way hydraulic micromanipulator (Narishige, Tokyo, Japan) for placement of the tips on the cell membrane. High resistance seals (greater than l gigaohm) were established by applying a slight suction between fire polished pipette tips (3–l0 megohm) and cell membranes. The offset potentials between pipette and bath solution were corrected with an offset circuit before each experiment. Pipette potential was clamped and single-channel K_Ca_ currents were recorded from cell-attached patches as described below through a List EPC-7 patch clamp amplifier (List Biological Laboratories Inc, Campbell, CA, USA). The amplifier output was low-pass filtered at 1 kHz with an 8-pole Bessel filter (Frequency Devices, Haverhill, MA, USA). Current signals were digitized at a sampling rate of 2.5 kHz. Single-channel K_Ca_ currents were analyzed using a pClamp software package (Axon Instruments, Molecular Devices, Sunnyvale, CA, USA, pClamp versions 10.2) to determine mean unitary current amplitudes and open state probability (NPo) as previously described [19], [20].

### Patch Clamp Potassium Channel Current Recording Solutions

Recording pipette solution contained (in mM): KCl 145, CaCl_2_ 1.8, MgCl_2_ 1.1, HEPES 5, ethylene-glycol-bis (β-aminoethyl ether)-N, N, N’, N’-tetraacetic acid (EGTA) 5, with the final pH adjusted to 7.2 with KOH. During recording from cell-attached patches the bath solution was composed of (in mM): KCl 145, CaCl_2_ 1.8, MgCl_2_ 1.1, HEPES 5, EGTA 5, with pH adjusted to 7.2 with KOH. This resulted in a calculated final bath [Ca^2+^] of 10^−7^M [15], [20]. The unitary conductance of the single-channel K_Ca_ current native in freshly isolated cerebral arterial muscle cells was previously identified to be 238 pS [15]. To determine if the potentiating effect of treatment with the PTEN inhibitor on the pressure-dependent myogenic constriction of cannulated cerebral arterial segments is related to modulation of the activities of cerebral arterial K_Ca_ channel current, the influence of inhibition of endogenous PTEN activity with bpV(phen) or bpV(pic) (10 µM) was examined on the activities of a 238 pS single-channel K_Ca_ current recorded from cell-attached patches of cerebral arterial muscle cells at a patch potential of +40 mV using symmetrical KCl (145 mM) solutions. To determine if the effects of the PTEN inhibitor bpV(phen) or bpV(pic) on the opening probabilities of the 238 pS K_Ca_ single-channel current is linked to the PI3K/Akt pathway, we also examined if the effect of the PTEN inhibitor bpV(phen) or bpV(pic) were antagonized or reversed by prior inhibition of the PI3K/Akt pathway using a specific and potent inhibitor of the PI3K/Akt signaling pathway ZSTK474 (1 µM, [21], [22], [23])**.** The concentration and the optimal time of incubation for the PTEN inhibitor bpV(phen) or bpV(pic) was adopted from a previous study that characterized its optimal inhibitory actions on PTEN activity [24].

### Intracellular Calcium [Ca^2+^]_i_ Measurements

The ratiometric fluorescent dye Fura-2-acetoxymethyl ester (Fura-2AM) was used to measure [Ca^2+^]_i_ in freshly isolated rat cerebral arterial muscle cells. Briefly, freshly isolated rat cerebral arterial muscle cells by enzymatic digestion were allowed to adhere on a 35-mm glass bottom petri dishes and loaded with Fura-2 AM (5 µM) at room temperature for 30–60 min in modified Hank’s Balanced Salt Solution (HBSS) that contained (in mM): 130 NaCl, 5.4 KCl, 1.6 CaCl_2_, 0.5 MgCl_2_, 0.4 MgSO_4_, 4.2 NaHCO_3_, 0.3 Na_2_HPO_4_, 0.4 KH_2_PO_4_, 5.5 glucose, and 20 HEPES (pH 7.4). The adhering cerebral arterial muscle cells were washed and incubated in fresh HBSS for an additional 15–30 min to allow complete deesterization of the dye. Fluorescence images with or without pretreatment with the PTEN inhibitor bpV(phen) or bpV(pic) (at 10 or 30 µM) and in the absence or presence of the specific PI3K/Akt signaling pathway inhibitor ZSTK474 (1 µM) were captured and analyzed using an image system consisting of an inverted epifluorescence microscope (Nikon TE200) with a 20× fluor objective, a high-speed wavelength switcher (Lambda DG-4 from Sutter Instruments), a PC-controlled digital charge-coupled device camera (Hamamatsu C4742-95), and Metafluor software (Universal Imaging) as previously described [25]. Fura-2 was excited alternatively at two wavelengths of 340 and 380 nm, and the emitted light was collected at an emission wavelength of 510 nm. Fura-2 fluorescence was acquired every 3 s. Results are presented as the ratio of the fluorescence intensity at 340 nm vs. 380 nm excitation (F340/F380). In some of the studies results are expressed as a change in fluorescent ratio from base line values. All experiments were performed at 37°C.

### Statistical Analysis

Data are expressed as mean ± SE. Differences between mean values were assessed using a Student’s t-test or analysis of variance (ANOVA) for multiple comparisons followed by a Duncan’s new multiple range tests. A P value of less than 0.05 was considered statically significant.

## Results

### Production of ROS in Pressurized Isolated Cerebral Arterial Segment

**Figure 1:**
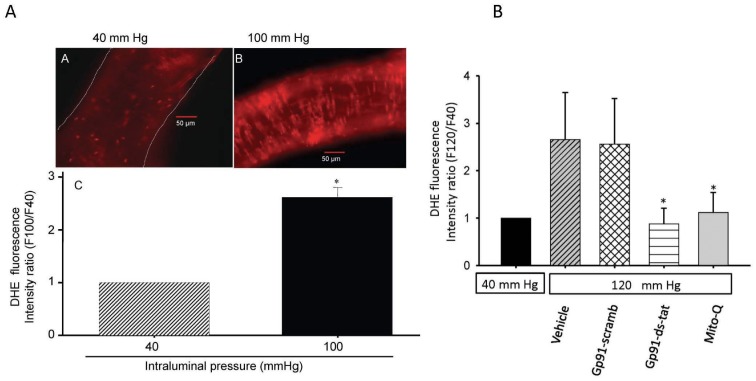
Elevation of intravascular pressure induced changes in superoxide production as determined by fluorescent microscopy analysis. (I) An increase in intravascular pressure induced increased production of O_2_^–^ in cannulated rat cerebral arterial segments as revealed by a significant increase in the fluorescent intensity ratio of dihydroethidium (DHE) fluorescence (A vs B). The magnification in panel A or B is 200×. Panel C depicts bar graphs comparing the DHE fluorescence intensity ratio following pressurization of cannulated cerebral arterial segments at 40 and 100 mm Hg. The increase in intraluminal pressure from 40 to 100 mm Hg significantly increased the DHE fluorescence intensity ratio as summarized in panel C. *P<0.05, n = 3–5 independent trials. (II) in further studies, pressurization of the cannulated cerebral arterial segments from 40 mm Hg to 120 mm Hg markedly increased the DHE fluorescence intensity ratio, which was significantly attenuated by prior treatment with the peptide inhibitor of NADPH-oxidase gp91-ds-tat (5 µM) or with the mitochondrial antioxidant MitoQ (1 µM), whereas pretreatment with the control gp91-scarmbled-tat peptide had no effect. These findings indicate that NADPH oxidase and the mitochondria are the possible sources for the pressure induced ROS production (n = 4–5 independent studies for each group, *denote significant difference from control at P<0.05).

**(i) Fluorescence microscopy detection of O_2_^–^ generation in pressurized cerebral arterial segments.** To examine the capacity of an increase intraluminal pressure to induce generation of superoxide, cerebral arterial segments were cannulated and pre-incubated with the fluorescent superoxide detecting probe DHE (1 µM) for 20 min in the absence or presence of the specific peptide inhibitor of NADPH-oxidase gp91ds-tat (5 µM) or its control scrambled gp91 sequence (gp91-scramb-tat, 5 µM), or with the mitochondrial antioxidant MitoQ (1 µM). As depicted in Fig. 1 A and B a step increase in intraluminal pressure from 40 mm Hg to 100 mm Hg or 120 mm Hg induced a significant pressure-dependent increase in the fluorescence intensity ratio of 2-OH-E^+^, the oxidation product of DHE by O_2_^–^. This increase in the intensity of 2-OH-E^+^ fluorescence was significantly attenuated by prior treatment with the peptide inhibitor of NADPH-oxidase gp91ds-tat (5 µM) or with the mitochondrial antioxidant MitoQ (1 µM), whereas it was not affected by treatment with the control gp91-scrambled-tat peptide, indicating that both the NADPH-oxidase and mitochondria generated O_2_^–^ are possible sources for the increase in intraluminal pressure-induced O_2_^.−^ production in the pressurized cerebral arterial segments. These findings indicate that an increase in intraluminal pressure from 40 mm Hg to 100 mm Hg or 120 mm Hg induces a significant increase in the intensity ratio of the DHE oxidation product 2-OH-E^+^ fluorescent signal (n = 5, *p<0.05).

**Figure 2:**
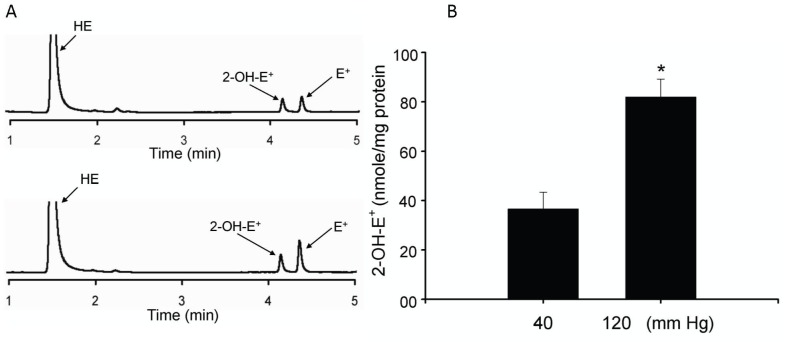
Fluorescence HPLC determination of the level of formation of O_2_^–^ in response to an increase in intraluminal pressure. The elevation in intraluminal pressure from 40 mm Hg to 120 mm Hg induced a significantly increased concentration of 2-OH-E^+^, the oxidation product of the superoxide detecting probe DHE by O_2_^–^, normalized to tissue protein (n = 4 separate experiments for each group, *denote P<0.05 compared to control level of 2-OH-E^+^ to that following the increase in intraluminal pressure to 120 mm Hg.

**(ii) Fluorescence HPLC detection of an increase in intraluminal pressure-induced superoxide production in cerebral arterial segments.** Using the recently developed fluorescence HPLC analysis for quantitation of O_2_^–^ production [15], [16], [17], [18], we examined if an increase in intraluminal pressure from 40 mm Hg to 120 mm Hg in cannulated cerebral arterial segments induces increased production of O_2_^–^ as determined by the level of formation of 2-hydroxyethidium (2-OH-E^+^), the oxidation product of DHE by O_2_^.−^ using the HPLC fluorescent assay method. As depicted in Fig. 2 A and B, the increase in intraluminal pressure from 40 mm Hg to 120 mm Hg in the cannulated cerebral arterial segments significantly increased the concentration of formed 2-OH-E^+^ normalized to mg of tissue protein (*p<0.02 n = 4–5) (Fig. 2B). These findings indicated that an increase in intraluminal pressure from 40 mm Hg to 120 mm Hg resulted in a significant increase in O_2_^–^ production in the cannulated cerebral arterial segments.

### Effect of Tempol, Mito-tempol and PEG-catalase on Intravascular Pressure-induced Myogenic Constriction of Cerebral Arteries

**Figure 3:**
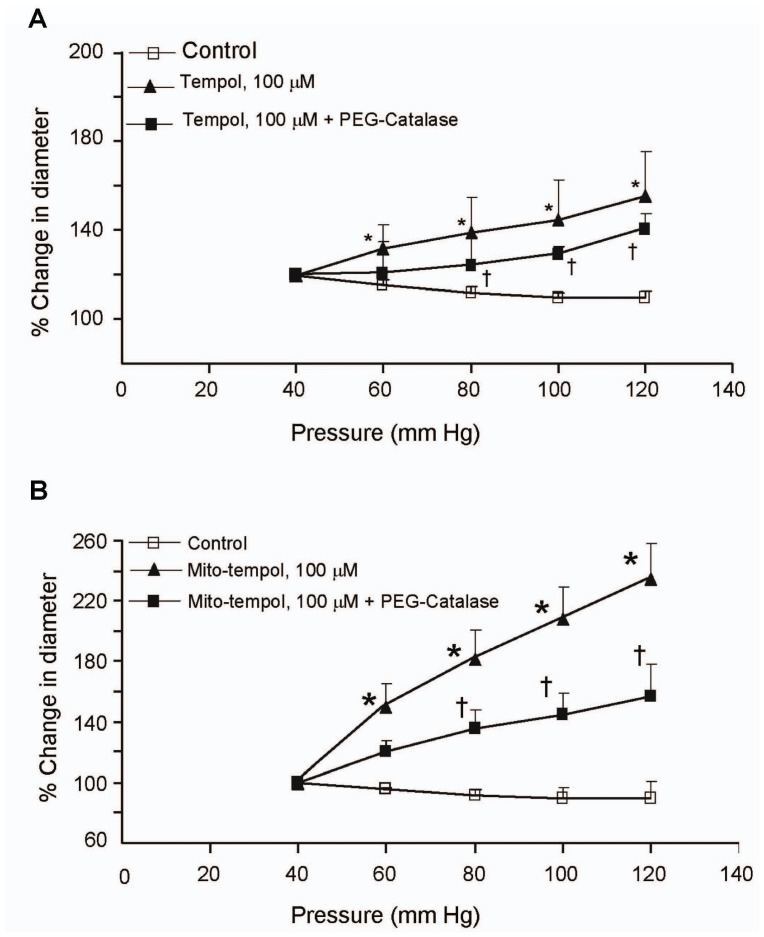
Dismutation of the ROS superoxide and H_2_O_2_ attenuated the increase in intraluminal pressure-induced myogenic constriction of cerebral arterial segments. Increasing intraluminal pressure over the range of 60 mm Hg to 120 mm Hg in steps of 20 mm Hg induced pressure-dependent myogenic cerebral arterial constriction that was significantly attenuated and converted to vasodilation by pretreatment of the cannulated pressurized cerebral arterial segments with the superoxide dismutase mimic tempol and tempol plus the H_2_O_2_ dismutase PEG-catalase (A) or with mitochondrial targeted mito-tempol and mito-tempol plus PEG-catalase (B). Data are presented as mean value ± SEM, n = 6–8 cerebral arterial segments per group. *P<0.05, **P<0.001.

The sources and role of ROS in regulating pressure-induced myogenic response was examined by determining the effects of tempol, a superoxide dismutase mimetic, mito-tempol, a mitochondrial-targeted form of tempol, and PEG-catalase. Rat isolated and cannulated cerebral arterial segments were mounted equilibrated for 60 min at 40 mm Hg. Under these conditions the cannulated arterial segments developed spontaneous tone that was equivalent to 47±2% of baseline diameter (184±8.4 µm). Treatment of the arterial segments with 100 µM tempol or 100 µM mito-tempol markedly attenuated the increase in pressure induced constriction of cerebral arterial segments (Fig. 3 A and B). Furthermore, treatment with PEG-catalase (100 mU/ml) elicited a marked reduction in pressure-induced cerebral arterial constriction in the presence of tempol and mito-tempol (Fig. 3 A and B).

### Elevation in Intravascular Pressure Induces Oxidative Inactivation of PTEN

**Figure 4:**
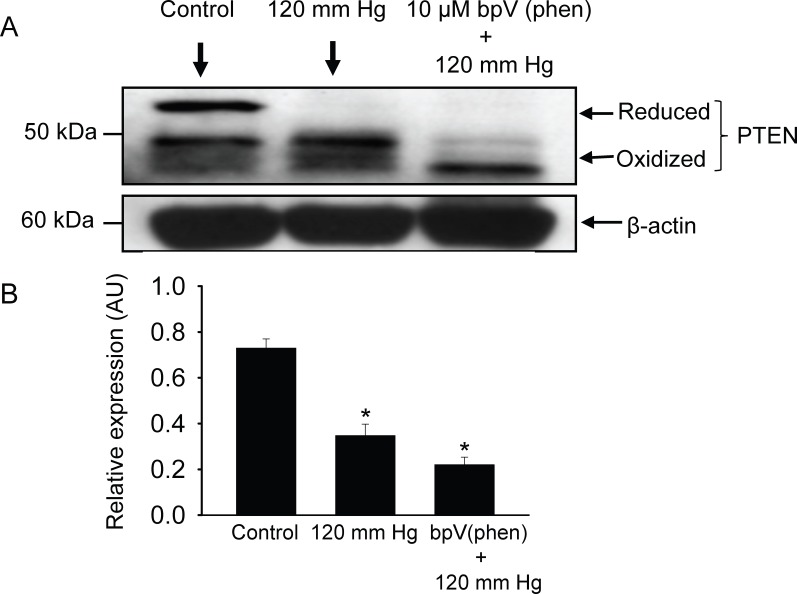
Pharmacological inhibition of endogenous PTEN activity potentiates an increased intravascular pressure induced inactivation of endogenous PTEN activity. (A) An increase in intravascular pressure to 120 mm Hg induced oxidation and inactivation of PTEN, and treatment of cannulated and pressurized cerebral arterial segments with the PTEN inhibitor bpV(phen) (10 µM) for 30 min caused a significant enhancement of the oxidation inactivation of PTEN evoked by an increase in intravascular pressure to 120 mmHg for 60 minutes. n = 3 independent experiments. (B) Bar graphs depicting summary of the density of expression level the PTEN protein in response to an increase in intraluminal pressure to 120 mm Hg before and after treatment with the PTEN inhibitor bpV(phen) (10 µM). *denotes significant difference from the control at p<0.05, n = 3 independent trials.

**Figure 5:**
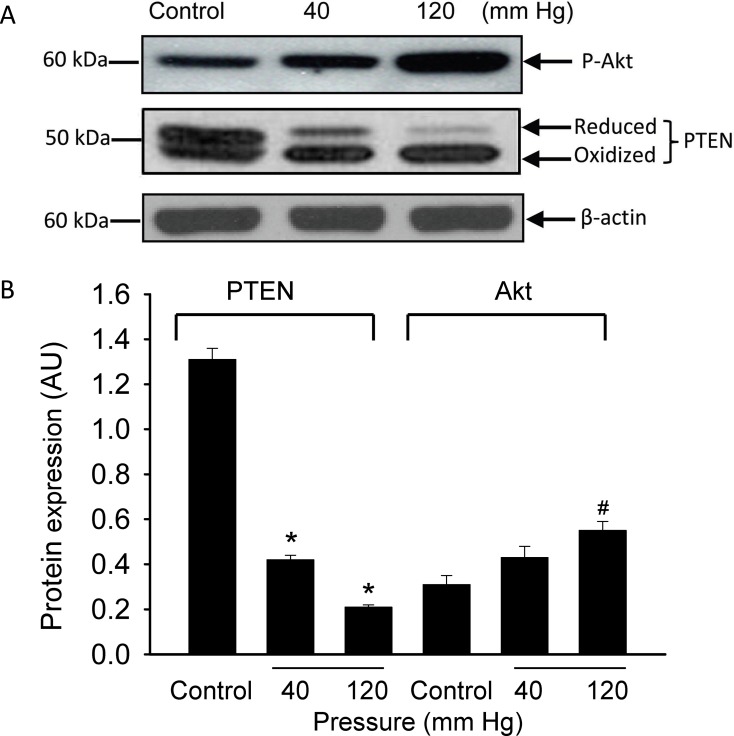
Effect of increasing intraluminal pressure in cannulated cerebral arterial segments on the activity levels of the phosphatase PTEN and the kinase Akt. Pressurization of the cannulated cerebral arterial segments at 120 mm Hg for 60 minutes exhibited a significant oxidative inactivation of PTEN, whereas this increase in intraluminal pressure induced elevated level of phosphorylated Akt as revealed by the change in density of the expression level of the protein bands for PTEN and phospho-Akt. Bar graphs depict summary of the density of the protein bands presented as mean ± SEM for the oxidized and reduced forms of PTEN and for that of phospho-Akt. n = 2–3 trials per group. *P<0.01, ^#^ P<0.05 compared to control group.

Pressurization of the arterial segments for 40 min at 120 mmHg resulted in the appearance of a higher mobility (oxidized) form of PTEN protein band that was further reduced in size in the presence of the PTEN inhibitor bpV(phen) (10 µM) (Fig. 4 A and B). These findings indicated that PTEN is oxidized and inactivated in response to an increase in intraluminal pressure or pharmacological inhibition of its activity, which could partly be attributed to the effects of an increase in intraluminal pressure-induced generation of the ROS O_2_^–^ and H_2_O_2_ as demonstrated in the present study. Since inhibition of or inactivation of PTEN would shift the balance between phosphatidylinositol (PI)-triphosphate (PIP_3_) and PI-biphosphate (PIP_2_) toward accumulation of PIP_3_ and result in phosphorylation or activation of the downstream kinase Akt, we also examined the phosphorylation level of Akt in response to an increase in intraluminal pressure from 40 to 120 mm Hg. As could be seen in Fig. 5 A and B elevation in the intraluminal pressure from 40 mm Hg to 120 mm Hg induced an increase in the formation of phosphorylated Akt.

### Inhibition of Endogenous PTEN Potentiates or Enhances Intravascular Pressure-induced Myogenic Constriction of Cerebral Arteries

**Figure 6:**
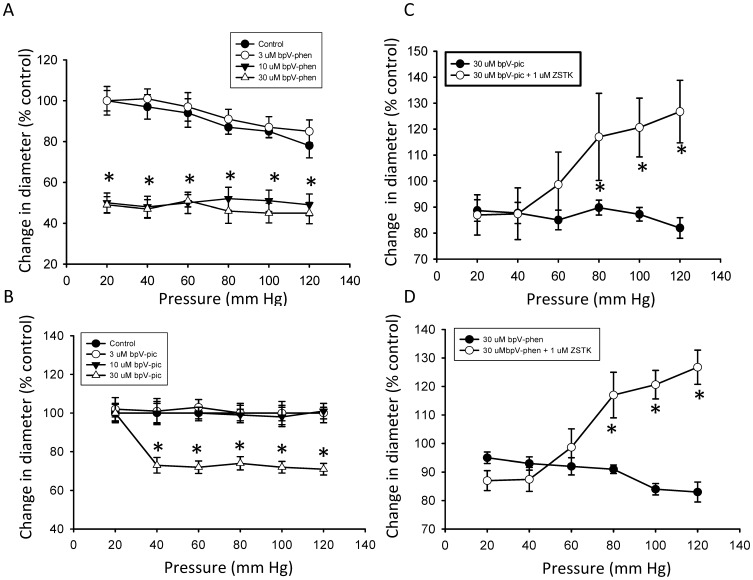
Pharmacological inhibition of endogenous PTEN activity enhances elevation in intraluminal pressure induced myogenic cerebral arterial constriction. Pretreatment of cannulated and pressurized cerebral arterial segments with the phosphatase PTEN inhibitor bpV(phen) (at concentrations of 3,10 or 30 µM) (A) or bpV(pic) (at concentrations of 3,10 or 30 µM) (B) for 30 min over intraluminal pressure range of 20 to 120 mm Hg in 20 mm Hg steps caused concentration-dependent enhancement of the increase in intraluminal pressure-induced myogenic constriction. n = 4–5 independent experiments. *and † denote significant difference at P<0.05. The PTEN inhibitor bpV(phen) induced potentiation of the myogenic response that reached maximum starting at 10 µM, whereas the PTEN inhibitor bpV(pic) appeared to elicit enhamcement of the pressure-dependent myogenic tone at 30 µM.

**Figure 7:**
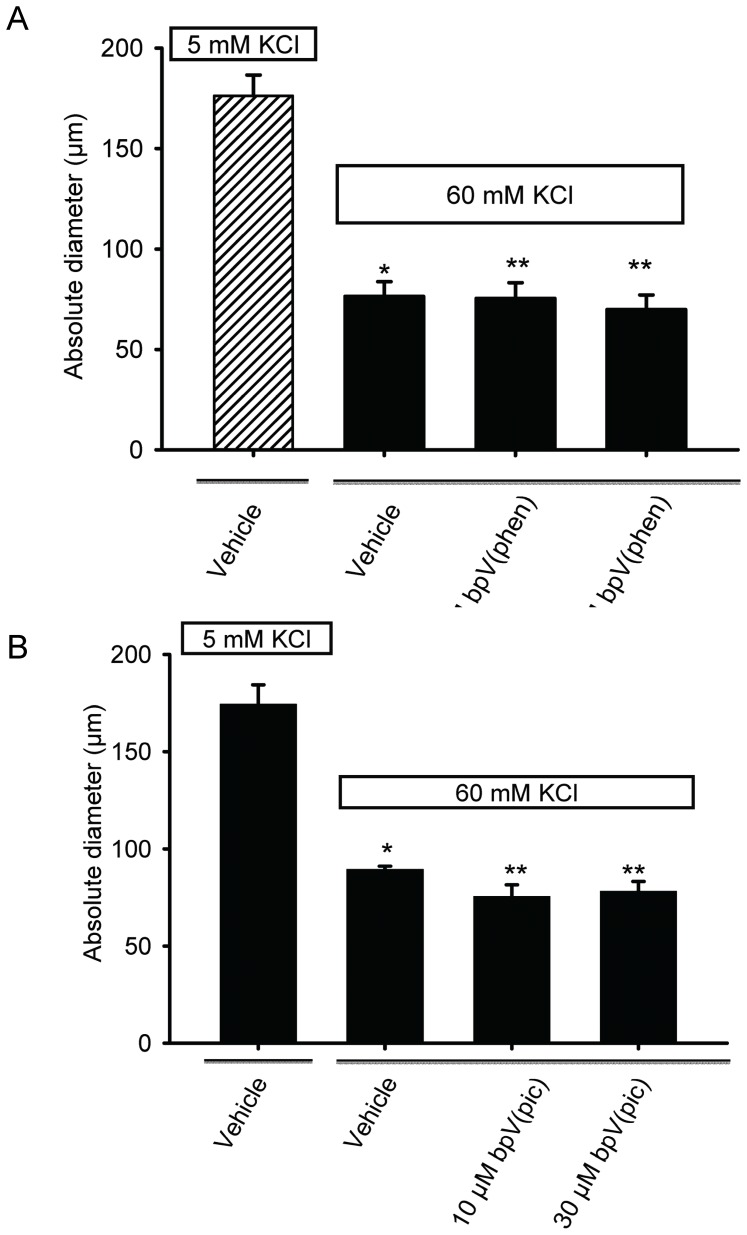
Inhibition of endogenous PTEN activity had no effect on high KCl (60 mM) induced cerebral arterial constriction. The high KCl (60 mM) induced constriction of pressurized (40 mm Hg) cerebral arterial segments was not influenced by pretreatment with either of the PTEN inhibitors bpV(phen) (10 µM or 30 µM) (A) or bpV(pic) (10 µM or 30 µM) (B). *denotes significant difference from control at p<0.05; n = 4–5 independent studies.

Studies were initiated to examine if the dual phosphatase PTEN, which is known to be oxidized and inactivated by H_2_O_2_ [26], [27]_,_ plays a negative feedback regulatory role in the development of pressure-dependent myogenic constriction of cannulated rat cerebral arterial segments. Indeed, as depicted in Fig. 6 A and B, pretreatment of cannulated cerebral arterial segments with two structurally different PTEN inhibitors bpV(phen) (3, 10, 30 µM) and bpV(pic) (3, 10, 30 µM) separately for 30 min induced a concentration-related enhancement of pressure-dependent myogenic constriction of cannulated cerebral arterial segments that reached maximal at 10 or 30 µM during elevation of intravascular pressure from 20 mm Hg to 120 mm Hg in steps of 20 mm Hg despite bpV(pic) appeared to be less efficacious in inducing such effect. However, this potentiating effect of inhibition of PTEN activity is found to have no effect on the non-specific high KCl (60 mM) induced constriction of pressurized cerebral arterial segments (Fig. 7 A and B), indicating that the potentiating action of inhibition of PTEN activity may be regarded to be specific for the pressure-induced myogenic constriction, which in the present study demonstrated to be associated with increased production of ROS. This PTEN inhibition evoked potentiation of pressure-dependent myogenic cerebral arterial constriction was attenuated in the presence the PI3K/Akt pathway inhibitor ZSTK474 (1 µM) [21], [22], [23]. This new finding suggests that under normal physiological conditions PTEN could regulate the magnitude of pressure-induced myogenic constriction by exerting a negative feedback type influence on PI3K, which could be tuned by the level of pressure–induced production of the oxidants O_2_^–^ and H_2_O_2._ These findings may indicate the involvement of the PI3K/PTEN axis as a signaling pathway in the modulation of pressure-induced myogenic cerebral arterial constriction under physiological or pathophysiological conditions associated with elevated oxidant production.

### Effects of Inhibition of Endogenous PTEN on the Open State Probabilities of Single-channel K_Ca_ Currents in Isolated Rat Cerebral Arterial Muscle Cells

**Figure 8:**
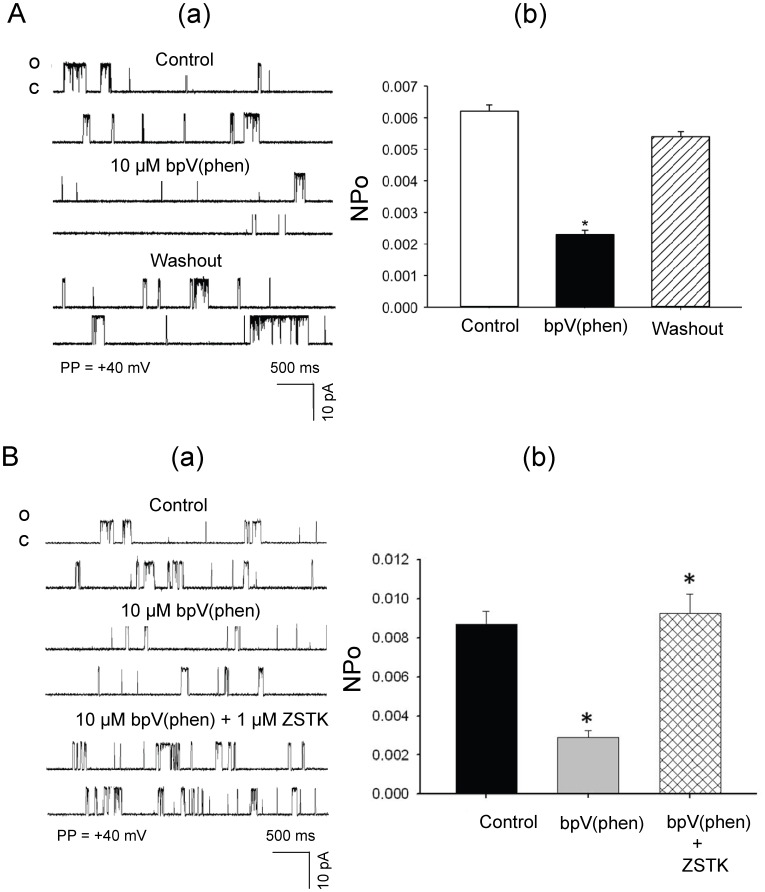
Inhibition of PTEN activity reduces whereas inhibition of PI3K increases the openings of a 238 pS K_Ca_ channel current in cerebral arterial muscle cells. (A) Effects of inhibition of endogenous PTEN activity on the open state probability (NPo) of a 238 pS single-channel K_Ca_ in isolated rat cerebral arterial muscle cells: (a) treatment of cell-attached patches of isolated cerebral arterial muscle cells with the PTEN inhibitor bpV(phen) (10 µM) markedly reduced (within 2–5 min) the opening frequencyies of the single-channel K_Ca_ currents recorded at a patch potential of +40 mV using symmetrical (145 mM) KCl recording solution without affecting the single channel amplitude. This effect of the PTEN inhibitor on the openings of the K_Ca_ single-channel current returned to control level following washout. (b) Bar graphs showing the inhibitory effects of application of the PTEN inhibitor bpV(phen) (10 µM) to the bath on in the mean NPo of the 238 pS single-channel K_Ca_ current in cell-attached patches of cerebral arterial muscle cells that were reversed following washout. *denotes p<0.05, n = 5–6 cells. (B) Treatment of the cell-attached patches with the PI3K inhibitor ZSTK474 (1 µM) significantly prevented or attenuated the PTEN inhibitor bpV(phen) (10 µM) induced reduction in the opening frequency (panel a) and NPo (panel b) of the 238 pS K_Ca_ single channel current (n = 4–5 cells for each group, *p<0.05).

As depicted in Fig. 8 A and B, treatment with the PTEN inhibitor bpV (phen) (10 µM) by addition to the bath induced a significant reduction in the opening frequency and open sate probability (NPo) of the 238 pS K_Ca_ single-channel current recorded at a patch potential of +40 mV from cell-attached patches of freshly isolated cerebral arterial muscle cells (CAMCs) bathed in symmetrical KCl (145 mM) recording solution [15]. Thus, the NPo values of the 238 pS single-channel K_Ca_ current averaged 0.0065±0.0003 under control conditions and 0.0022±0.0004 after treatment with 10 µM bpV(phen) and 0.0068±0.00071 following washout (Fig. 8 A, p<0.05, n = 6 cells). The PTEN inhibition induced reduction in NPo and opening frequency of the 238 pS K_Ca_ single-channel current were prevented or reversed following treatment of the patch-clamped cerebral arterial muscle cells with the PI3K inhibitor ZSTK747 (1 µM). In these studies the PTEN inhibitor induced reduction in NPo was significantly increased following inhibition of the PI3K/Akt pathway with ZSTK474 (0.0031±0.00013 before and 0.0093±0.000125 after, p<0.05, n = 4). Taken together, these findings may suggest that a balance in activity between endogenous PTEN and the PI3K pathway regulates basal activities of single-channel K_Ca_ currents in rat cerebral arterial muscle cells. These findings may also further indicate that increased PI3K activity, in situations where inactivation of PTEN occurs, imposes an inhibitory influence on the open state probability of the cerebral arterial K_Ca_ channel current that could be linked to activation of the downstream kinase Akt enabled phosphorylation of a variety of proteins including the K_Ca_ channel subunits, which could lead to membrane depolarization. These findings could be regarded to suggest that inactivation of PTEN activity could reduce openings of arterial K_Ca_ channel current through stimulation of the PI3K/Akt signaling pathway that may lead to membrane depolarization-dependent Ca^2+^ influx and rise in intracellular calcium.

### Effects of Inhibition of the Phosphatase PTEN Activity on [Ca^2+^]_i_ Levels in Freshly Isolated Rat Cerebral Arterial Muscle Cells

**Figure 9:**
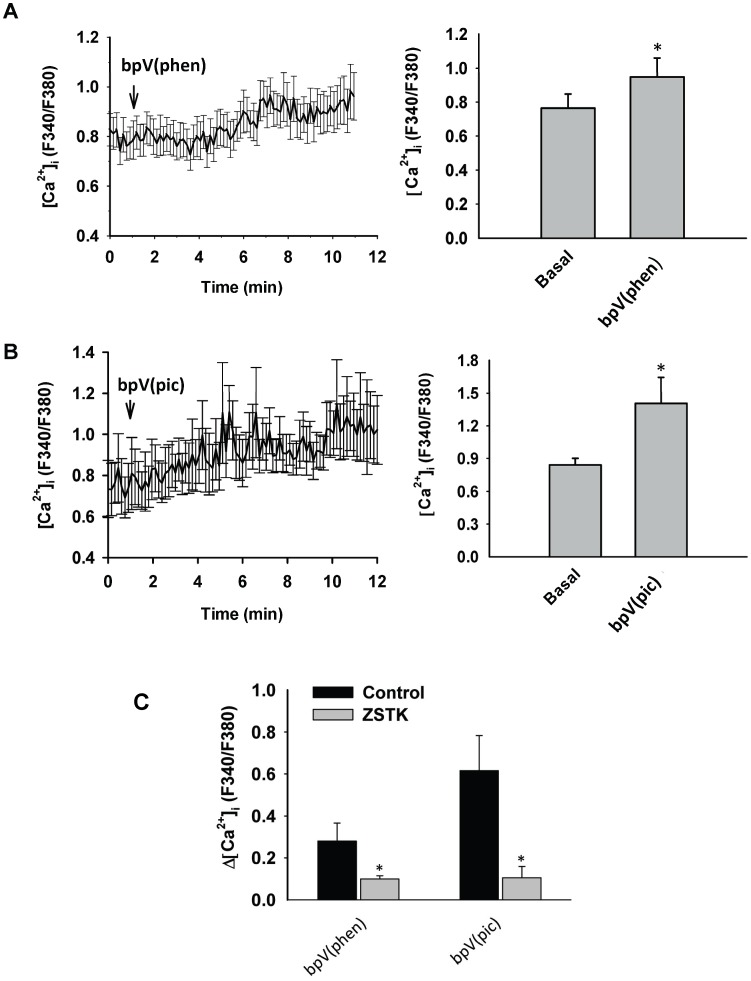
Effects of the specific PTEN inhibitor bpV(phen) (10 µM) or bpV(pic) (10 µM) on [Ca^2+^]_i_ response of Fura-2AM loaded freshly isolated cerebral arterial muscle cells (CAMCs). Treatment of the Fura-2AM loaded CAMCs with 10 µM bpV(phen) (A) or with 10 µM bpV(pic) (B) caused a significant increase in [Ca^2+^]_i_ that developed over time. Bar graphs shown on the right of panel A or panel B depict summary of the ratio of the fluorescent calcium signals indicating a significant increase in the ratio of [Ca^2+^]_i_ in response to treatment with the PTEN inhibitor bpV(phen) (10 µM) or 10 µM bpV(pic) as compared to the respective controls. n = 5–6 independent experiments for each group. *denotes significant increases in [Ca^2+^]_i_ at p<0.05. (C) bar graphs depicting the ability of the PI3K inhibitor ZSTK474 (1 µM) to significantly attenuate or prevent the PTEN inhibitor bpV (phen) (10 µM) or bpV (pic) (10 µM) induced increase in [Ca^2+^]_i_ expressed as change in the ratio of [Ca^2+^]_i_ in CAMCs loaded with Fluor-2AM. *denote significant difference from control at p<0.001; n = 4 to 5 independent experiments.

The effects of treatment with two structurally different specific PTEN inhibitors bpV(phen) (10 µM) and bpV(pic) (10 µM) on the level of intracellular calcium in freshly isolated CAMCs loaded with the fluorescent calcium detecting probe Fura-2AM were investigated. Treatment of CAMCs with the PTEN inhibitor bpV(phen) (10 µM) caused an increase in [Ca^2+^]_i_ (F340/F380∶0.76±0.08 basal vs. 0.94±0.114 after treatment with bpV (phen), p<0.05, n = 6 independent assays, whereas treatment of the CAMCs with 10 µM bpV (pic) induced an increase in [Ca^2+^]_i_ (F340/F380∶0.84±0.06 basal vs. 1.14±0.30 after treatment with bpV (pic), p<0.012, n = 4 independent assays (Fig. 9 A and B), which reached maximum level over time for both treatment conditions. In additional studies, pretreatment of the Fura-2AM loaded CAMCs with the specific PI3K/Akt pathway inhibitor ZSTK474 (1 µM) significantly blunted the increase in [Ca^2+^]_I_, expressed as a change in fluorescence ratio from respective basal values, induced by either 10 µM bpV (phen) (0.28±0.11 before and 0.11±0.02 after; p<0.05, n = 4–6 independent assays) or by (10 µM) bpV (pic) (0.61±0.16 before and 0.13±0.07 after; *p<0.05, n = 4–5 independent assays) (Fig. 9 C). This findings may indicate that oxidative inactivation of PTEN, that could occur during an increase in intraluminal pressure or in a conditions of oxidative stress associated with elevated levels of the oxidants O_2_^–^ and H_2_O_2_, may act to increase [Ca^2+^]_i_ levels that could lead to enhanced vasoconstriction through activation of the PI3K/Akt pathway.

## Discussion

In the present study we found that an increase intraluminal pressure in cannulated cerebral arteries induces elevated production of the ROS superoxide (O_2_^–^) and oxidative inactivation of the dual phosphatase PTEN. The results obtained also demonstrated that inhibition of endogenous PTEN activity induces a reduction in NPo of a 238 pS K_Ca_ single-channel current, an increase in intracellular concentration of Ca^2+^ ([Ca^2+^]_i_) in isolated cerebral arterial muscle cells, and an enhancement of pressure-induced myogenic cerebral arterial constriction-responses that were significantly attenuated following inhibition of the PI3K/Akt pathway.

Given the existence of a negative feedback type relationship between the activities of the phosphatase PTEN and PI3K [11], [12], [13], [14], inhibition or inactivation of PTEN could be expected to result in upregulation or stimulation of PI3K and its downstream target Akt that could serve as a signaling mechanism mediating the cellular effects of inactivated PTEN. Interestingly, such possible signaling role of the PI3K/Akt pathway is confirmed by the findings of the present study that demonstrated attenuation of the inhibition of PTEN induced reduction in K_Ca_ channel current activity, an increase in [Ca^2+^]_i_ and enhancement of the pressure-induced myogenic constriction by pharmacological inhibition of the activities of the PI3K/Akt pathway in cerebral arterial muscle. These findings also revealed existence of contrasting type functional interactions between the activities of PI3K and the phosphatase PTEN, which could have intriguing physiological and therapeutic relevance in the cerebral circulation consistent with what has been reported in the literature [28], [29].

It is possible that other endogenous signaling mechanisms that could be sensitive to oxidant action or an increase in intraluminal pressure or stretch, might also contribute to the inactivation of PTEN induced reduction in NPo and a rise in [Ca^2+^]_i_ observed under the experimental settings of the present study. Despite such possibilities, the findings of our present investigations revealed the existence of an increase in intraluminal pressure induced redox-sensitive signaling event triggered by the oxidative inactivation of the phosphatase PTEN that could influence pressure-dependent myogenic tone development under physiological or pathophysiological conditions associated with altered levels of ROS production.

In the present study pretreatment of isolated and cannulated middle cerebral arteries with the superoxide dismutase mimic tempol or mito-tempol alone or together with PEG-catalase resulted in a loss of pressure-induced cerebral vasoconstriction suggesting that mitochondrial sources of ROS generation also play an important role, and that the generated O_2_^–^ or H_2_O_2_ contributes to the development of the pressure-induced myogenic vasoconstriction. This conclusion is further bolstered by our observation that treatment with PEG-catalase converted the pressure-induced myogenic vasoconstriction to pressure-dependent vasodilation albeit to a lesser extent than that observed with the superoxide dismutase mimic tempol or mito-tempol alone (Fig. 3). In contrast to the present observations, however, a previous study [30] has shown that the effect of treatment with the superoxide dismutase mimic tempol was to potentiate pressure-induced myogenic constriction. The exact reason for the disparity in the action of superoxide dismutase mimic tempol between this previous study and our present finding is not known, but could be due to differences in anatomical origin of the arteries studied (gracilis arteries versus cerebral arteries) or differences in the experimental conditions employed (intermittent hypoxia versus normoxia). In regards to the findings reported in the present study, it is unclear what mediates the component of the pressure-induced cerebral arterial vasodilation that resulted following treatment with tempol or mito-tempol in the presence or absence of PEG catalase. Nevertheless, the most likely explanation for the observed evolution of this vasodilatory response could be possible recovery of oxidative inactivation of PTEN activity following scavenging of hydrogen peroxide released from dismutation of superoxide generated in response to elevation of intraluminal pressure.

**Figure 10:**
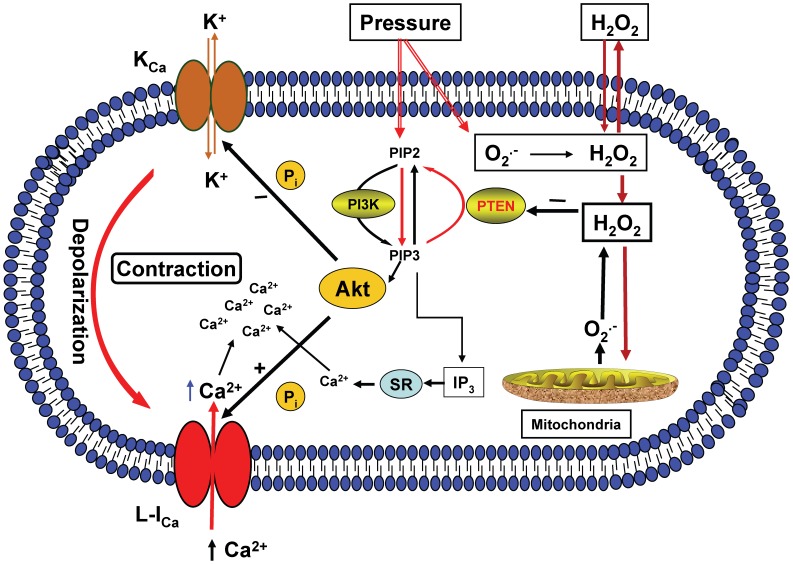
Schematic depicting conceptual presentation of consequences of an increase in intraluminal pressure-induced production of the ROS O_2_^–^ and H_2_O_2_ on cellular signaling events. The increase in intraluminal pressure-induced O_2_^–^ and H_2_O_2_ production triggers a redox sensitive signaling event via oxidative inactivation of the lipid phosphatase PTEN that induces activation of PI3K resulting in generation of phosphatidylinositol (PtdIns) (3,4,5,)P3 (PIP3) and release of IP3 and activation and recruitment of Akt, which could cause phosphorylation and inhibition of K_Ca_ channel activity, membrane depolarization and activation of L-type Ca^2+^ channel (L-I_Ca_), which together with the released IP3 increase intracellular Ca^2+^ level required to evoke pressure-dependent cerebral arterial myogenic constriction.

The precise signaling mechanism effecting the pressure-dependent cerebral arterial myogenic constriction in response to pressure-induced increased O_2_^–^ and H_2_O_2_ production is not known. However, based on the findings of the present studies it is possible to propose that the superoxide generated during an increase in intraluminal pressure is rapidly dismutated to H_2_O_2_ that could act to oxidize and inactivate the lipid phosphatase PTEN [27] native in cerebral arterial muscle that could result in up regulation of PI3K activity, release of inositol (3,4,5)-triphosphate (IP3) and increased formation or activation of the downstream molecule Akt or PKB [28], [29], [31]. The increased formation of Akt or PKB has the capacity to phosphorylate a number of proteins including the alpha-subunits of the arterial K_Ca_ channel and the arterial L-type Ca^2+^ channel, whereas the released IP3 stimulates release of Ca^2+^ from the sarcoplasmic reticulum (SR). As summarized in the conceptual schematic presentation depicted in Fig. 10, the phosphorylation facilitated by the activation of Akt or PKB could lead to inhibition of the activities of K_Ca_ channel, as revealed indirectly by the increased openings of the K_Ca_ channel following inhibition of the PI3K activity in the present study, leading to arterial muscle cell membrane depolarization that could activate voltage-dependent L-type Ca^2+^ channel mediated calcium entry into the arterial muscle cell, which is requisite for the development of pressure-induced myogenic constriction.

From the findings of the present studies we propose that pressure-induced increased production of ROS could serve as a surrogate to initiate a redox signaling mechanism enabled through oxidative inactivation of the lipid phosphatase PTEN, which could potentiate the increase in intraluminal pressure-induced myogenic constriction of rat cerebral arteries. To our knowledge such a potentiating action of inactivation of endogenous PTEN by oxidants generated in response to elevated intraluminal pressure on pressure-induced myogenic constriction has not been previously reported, and appears to be a novel mechanism through which an oxidized state of the lipid phosphatase PTEN imposes its action to enhance pressure-induced cerebral arterial myogenic constriction by increasing the availability of activator Ca^2+^, which might be exacerbated in conditions of oxidative stress where the level of ROS is expected to be very high. We presume that such oxidative inactivation of the phosphatase PTEN induced enhanced pressure-induced cerebral arterial myogenic contractile response, might be expected to serve as a physiologically beneficial vasoconstrictor influence that could ameliorate tissue damage resulting from possible over perfusion in ischemic conditions of the brain that are known to be associated with impaired autoregulation CBF [32], [33], [34].

There is controversy in the literature with regards to effects of oxidative inactivation of PTEN by H_2_O_2_ as related to the actions of H_2_O_2_ on the activities of membrane K_Ca_ channel currents. For example, in HEK 239 cells in which the K_Ca_ α-subunit (mslo), in the absence or presence of hβ_-_subunit, co-transfected with wild type PTEN (PTEN_WT_) was observed to reduce the H_2_O_2_-induced activation of K_Ca_ channel current, whereas co-transfection with the catalytically inactive mutants of PTEN, that lack phosphatase activity, was found to have no regulatory effects on the effects of H_2_O_2_ on K_Ca_ channel currents [35]. These investigators proposed the involvement of PTEN activity as a new mechanism for H_2_O_2_-induced K_Ca_ channel activation and thus for H_2_O_2_–induced vasodilatation [35]. Other investigators using a combination of biochemical and electrophysiological techniques demonstrated that oxidative inactivation of PTEN acts to enhance voltage-gated Ca^2+^ entry in cardiac myocytes that was mediated by an increase in PIP3 production and activation of the PI3-K/Akt pathway [36]. This later finding lead to the conclusion that inactivation of the phosphatase PTEN could protect the phosphorylated state of voltage-gated Ca^2+^ channels via upregulation of the PI3K/Akt pathway leading to enhancement of Ca^2+^ entry. The observations by the later investigators appear to indicate that basal activity of the phosphatase PTEN could be pivotal for homeostatic control of intracellular calcium level in different cell types, which could lend support for our present finding that demonstrated inhibition of endogenous PTEN-induced increased [Ca^2+^]_i_ level in cerebral arterial muscle cells. The observed disparity in the two previously reported actions of PTEN in two different cell types [35], [36] could be regarded as a reflection of differences in the actions or activities of PTEN in cultured HEK 239 cell lines versus native cardiac myocytes [35], [36]. Our present findings that oxidative inactivation of PTEN reduces the NPo of a 238 pS K_Ca_ single-channel current and promotes an increase in [Ca^2+^]_i_, in cerebral arterial muscle cells could, in part, be consistent with the reported findings in cardiac myocytes by Wan et al. [36], and support the notion that inactivation of PTEN could be regarded as one of the underlining mechanisms for pressure-induced cerebral arterial constriction, which our present studies demonstrated it to be associated with increased ROS production.

In conclusion, our present findings have revealed the existence of a novel redox signaling mechanism triggered by an increase in intraluminal pressure-induced ROS generation, namely oxidative inactivation of the phosphatase PTEN, which could mediate or modulate pressure-dependent cerebral arterial myogenic constriction via activation of the PI3K/Akt pathway, and might be crucial for the adjustment of nutritive cerebral blood flow to the brain under physiological or pathologic oxidative stress conditions to confer protection.
